# spammR: an R package designed for analysis and integration of spatial multi-omic measurements

**DOI:** 10.1093/bioadv/vbag163

**Published:** 2026-06-10

**Authors:** Yannick Mahlich, Harkirat Sohi, Marija Veličković, Paul D Piehowski, Jason E McDermott, Sara J C Gosline

**Affiliations:** Biological Sciences Division, Pacific Northwest National Laboratory, Richland, WA, United States; Biological Sciences Division, Pacific Northwest National Laboratory, Richland, WA, United States; Environmental and Molecular Sciences Division, Pacific Northwest National Laboratory, Richland, WA, United States; Environmental and Molecular Sciences Division, Pacific Northwest National Laboratory, Richland, WA, United States; Biological Sciences Division, Pacific Northwest National Laboratory, Richland, WA, United States; Department of Molecular Microbiology and Immunology, Oregon Health & Sciences University, Portland, OR 97201, United States; Biological Sciences Division, Pacific Northwest National Laboratory, Richland, WA, United States; Department of Biomedical Engineering, Oregon Healthy & Sciences University, Portland, OR 97201, USA

## Abstract

**Motivation:**

Spatial omics is a young and evolving field and as such shows rapid development of novel technologies and analysis methods to measure transcripts, proteins, metabolites, and post-translational modifications at high spatial resolution. These advances in technology have enabled the simultaneous generation of abundance profiles for multiple different omics types and associated microscopy imaging data, as well as their analysis in a spatial context. However, most analytical tools are designed for spatial transcriptomics platforms and are challenging to use in other contexts such as mass spectrometry-based measurements or metagenomics.

**Results:**

To this end we present spammR (spatial analysis of multi-omics measurements in R), an R package that enables end-to-end analysis with a specific focus on mass-spectrometry derived spatial omics datasets with the goal of integration across multiple data types (e.g. sequencing, metabolites, proteins) within the same tissue

**Availability and implementation:**

spammR is implemented in R. The package is currently installable from GitHub (https://pnnl-compbio.github.io/spammR/).

## 1 Introduction

The field of spatial biology (Crosetto *et al*. 2015, Moses and Pachter 2022, Vandereyken *et al*. 2023) and the growth of spatially-resolved technologies (Lundberg and Borner 2019, Taylor *et al*. 2021, Bouwman *et al*. 2022, Moffitt *et al*. 2022) has emerged from the need to study biological systems within their spatial context and native tissue environments (Kiessling and Kuppe 2024). Measuring the molecular changes of a sample, e.g. slices of tumor tissue, in its spatial context (Lee *et al*. 2014, Nichterwitz *et al*. 2018, Zhang *et al*. 2021), has been enabled by both novel transcriptomics (Chen *et al*. 2015, Codeluppi *et al*. 2018, Wang *et al*. 2018, Eng *et al*. 2019, Rodriques *et al*. 2019) as well as proteomics technologies (Angelo *et al*. 2014, Lin *et al*. 2018, Black *et al*. 2021, Radtke *et al*. 2022). Mass spectrometry (MS) based technologies to measure proteins (Zhu *et al*. 2018, Piehowski *et al*. 2020), lipids, metabolites, and other molecules (Greer *et al*. 2011, Buchberger *et al*. 2018, Veličković *et al*. 2024, 2025) have furthered enabled this field to study how molecular interactions can vary across a tissue.

While the pace of computational tool development has accelerated, particularly in the field of spatial transcriptomics (Walker *et al*. 2022), there is a gap in spatial analysis tools tailored towards the analysis and interpretation of mass spectrometry-based multi-omics data, with most existing tools focused on specific analytical frameworks (Nimo *et al.* 2025) that cannot be readily applied to integrative omics analyses–e.g. comparing metabolites and proteomics in the same sample. This is especially important regarding the distinct differences between antibody imaging and mass spectrometry-based measurement. For mass spectrometry specifically, those include (1) the need for normalization of mass/charge ratios, (2) the potential need to impute data to counteract missingness and, (3) MS as a technology not being restricted to proteomics. In addition to MS-based spatial proteomics, the rise of spatial glycomics, metabolomics, lipidomics and measurements of post translational modifications (PTMs), such as phosphorylation, all necessitate a more flexible framework than those that exist for other spatial technologies.

To this end, we introduce spammR (SPatial Analysis of Multiomics Measurements in R), an R package that is well-suited for an end-to-end analysis of mass-spectrometry derived spatial omics datasets with (1) smaller sample sizes and spatial sparsity of sampling, (2) considerable missingness, and (3) no a-priori knowledge about proteins or genes of interest, relying on a fully data-driven approach. Here we describe the overall features of spammR, and how it can be used in “traditional” spatial omics contexts, using proteomics data from pancreatic cancer, and lipidomics and proteomics data from rat brain as examples, as well as more broadly as a tool to perform comparative omics studies in a spatial context using metagenomic data from the 1000 soils project (Bowman *et al*. 2023, Song *et al.* 2025).

## 2 Implementation

**Figure 1:**
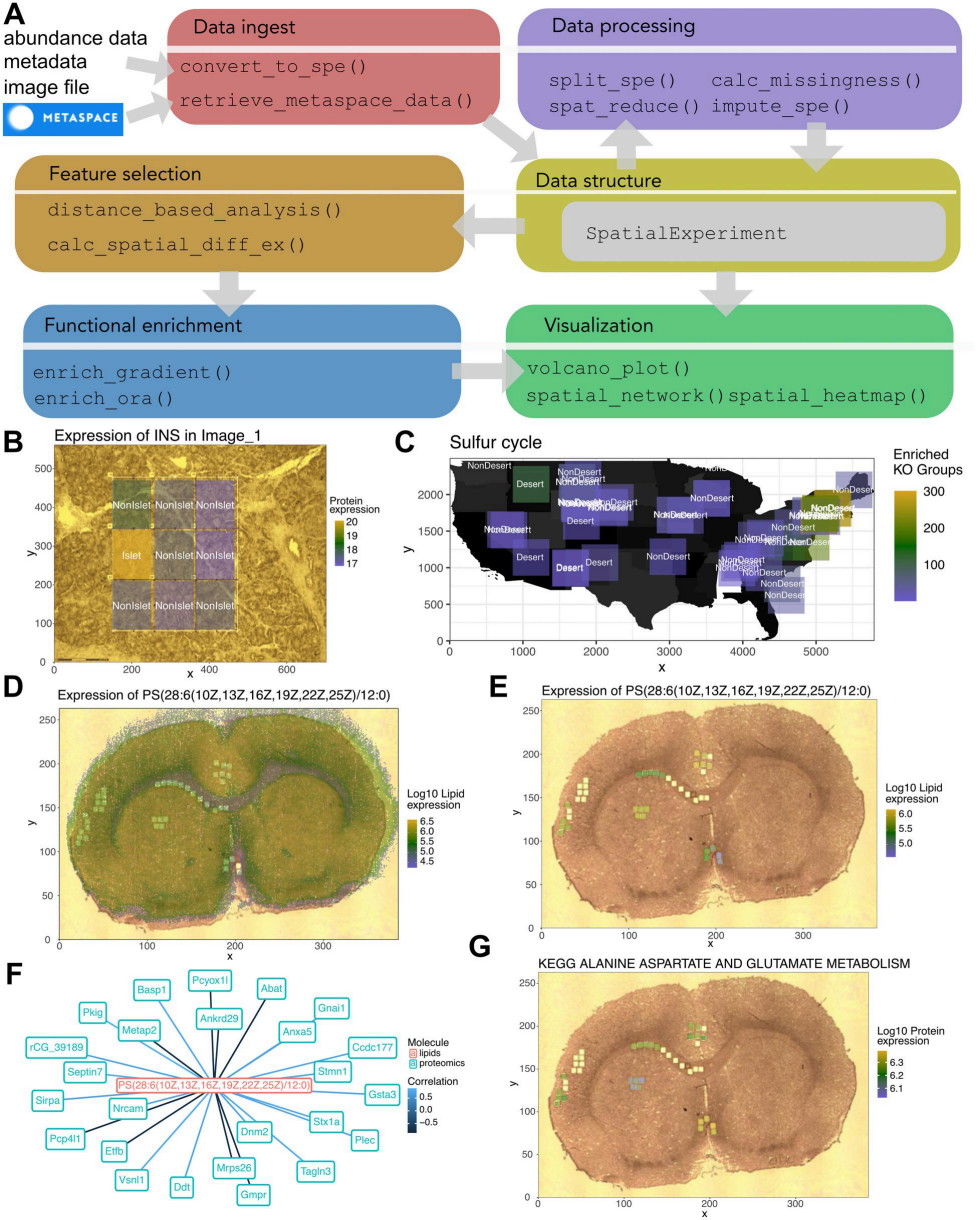
Overview of spammR functionality. (A) Flow chart describing use of spammR package as well as how the methods of the package relate to one another. (B) Expression of the protein insulin in a human pancreas tissue, with labels indicating whether the image region contains islet cells or not. (C) Abundance of the sulfur cycle KEGG orthology group across the US Soil study. (D) Expression of a highly variable lipid across a rat brain sample, as well (E) the expression of that lipid only within the regions measured by proteomics. (F) Network representing the lipid of interest (pink) and highly positively or negatively correlated proteins (turquoise). (G) Expression of proteins in the KEGG Alanine, aspartate, and glutamate pathway, which was identified as significantly enriched in proteins negatively correlated with the lipid of interest shown in panel.

spammR is a package implemented in R and will be available via Bioconductor. We make use of the ‘SpatialExperiment’ (Righelli *et al*. 2022) object via Bioconductor (Gentleman *et al*. 2004, Huber *et al*. 2015) to provide the ability to integrate with other tools in the Bioconductor environment, and for increased interoperability with other tools in the spatial omics field (Pardo *et al*. 2022, Feng *et al*. 2023, Windhager *et al*. 2023). The package consists of five main components (Fig. 1A): (1) Data ingest, (2) data processing, (3) feature selection, (4) functional enrichment, and (5) integrated visualization.

The data ingest component is comprised of two functions designed to capture the diverse data modalities that spammR analyzes. The primary function is convert_to_spe() and consumes three types of data: a data matrix of counts or values representing the samples measured (samples as columns, features as rows), a metadata table mapping the samples to a coordinate system as well as any other phenotypic data, and the image itself. spammR assumes that the molecular data are fully normalized/batch corrected (Välikangas *et al*. 2018), however additional corrections can also be applied depending on the data modality consumed (e.g. RNASeq vs proteomics). The function then returns a SpatialExperiment object containing the information that can be used for additional processing or analysis. To account for mass spectrometry imaging data on the Metaspace2020 platform (Veličković *et al*. 2021), we also included a retrieve_from_metaspace() function that selects a specific project and downloads both the coordinate metadata as well as the molecular quantifications. This function also returns a SpatialExperiment object. Each function only retrieves one data modality at a time, with merging happening using utility functions described below.

The data processing component currently consists of four functions designed to aid in multiomic analysis. To account for missingness in data, we included the calc_missingness() function to identify missing feature values in the object, and the impute_spe() that can run eight basic imputation algorithms. Another key feature of the spammR is the ability to measure diverse data modalities and samples. For this we included the spat_reduce() function that merges two SpatialExperiment objects on the same coordinate space as well as the split_spe() function that separates out a single object into multiple smaller objects.

The feature selection component contains functions to identify features (e.g. proteins) that either show strong correlation between abundances and spatial distance based on regions of interest (distance_based_analysis()) or display significant differential expression profiles based on sample categorization (e.g. a categorical distinction between ROIs) without incorporation of distance measures (calc_spatial_diff_ex()). Both functions take a SpatialExperiment object created by convert_to_spe() and return an augmented SpatialExperiment with the added significance rankings.

The functional enrichment component contains functions to generate over-representation statistics (ORA) for feature selection results. enrich_gradient() can be used to generate pathway enrichment based on results from the distance-based analysis (distance_based_analysis()). Analogously, enrich_ora() does the same for ranking results from categorical differential expression analysis. Both functions rely on leapR (Danna *et al*. 2021) for pathway and gene set enrichment analysis (GSEA) (Subramanian *et al*. 2005). Like the feature selection functions, both functions in the enrichment component will return an augmented SpatialExperiment object that can in turn be used for visualization of enriched pathways in ROIs.

Finally, the data contained in a SpatialExperiment object, either directly generated by the raw data ingestion or augmented using the feature selection components, can then be visualized using the visualization component which contains two different visualization functions. (1) spatial_heatmap() overlays a grid onto image data representing the different regions of interest (ROIs) and shading the individual grid cells by the calculated differential (e.g. raw abundance measures or differential expression calculated by one of the functional enrichment components). (2) volcano_plot() can be used to visualize the differential expression results generated by calc_spatial_diff_ex(). We also enable network-style visualizations through the spatial_network() function, which identifies pair-wise relationships between molecules across space that can then be plotted using the tidygraph R package.

All functions are designed to be lightweight, data agnostic, and provide interoperability via the SpatialExperiment object to enable usage beyond the functionality outlined here.

## 3 Application

We included in the spammR package three examples of applications using multiomics measurements across space.

### 3.1 Coordinate-based spatial heatmap in spammR enables contextual interpretation of spatial differences

To demonstrate the capabilities of spammR we conducted an analysis on previously published multi-omics data of human pancreatic tissue samples (Gosline *et al*. 2023). Specifically, we start by using the available methods in the spammR package to generate and visualize abundance differences for insulin across ROIs which predominantly contain islets or non-islet cells respectively, observing the expected behavior of higher abundance of insulin in islet grid cells (Fig. 1B). Next, we performed a pathway enrichment analysis using spammR’s feature selection and functional enrichment components by pooling images from seven distinct pancreas samples. As expected, pathways associated with insulin regulation and secretion are enriched islet cells in comparison to pancreatic regions containing predominantly non-islet cells. Finally, using the distance-based analysis functions in spammR we were able to identify and visualize the fact that AP3S2, a subunit of the AP-3 complex that is thought to be associated with protein transport (Cowles *et al*. 1997), expression strength is strongly anti-correlated with the distance from islet cells, i.e. decreasing expression with increasing distance. The entire analysis is showcased in one of the vignettes (spatProt.rmd) included with the spammR package.

### 3.2 spammR’s agnostic approach to multi-omics integration enables the investigation of metagenomic data in a geospatial context

The implementation of spammR’s spatial component is inherently agnostic to whether the distances within the investigated sample are on a micrometer, millimeter or even kilometer scale. To demonstrate this, we retrieved KEGG (Kanehisa *et al*. 2025) Ortholog (KO) presence data for 108 metagenome samples (54 sampling sites, samples each at two depths) from the 1000 soils project pilot study of MONet (Molecular Observation Network). Note that this is different from mass-spectrometry proteomics data in so far that in the 1000 soils data the number of predicted protein sequences from metagenome-assembled genomes (MAGs) that are associated with a given KO is recorded (i.e. presence), whereas traditional MS-proteomics data reports quantitative abundance measurements. This leads to significantly lower “abundance values” as well as lower variance between the individual values. In addition to the KO abundance, each sample contains further metadata, like soil temperature, water content, pH, metal abundances, and most importantly for spatial context, latitude, longitude, elevation and whether the sample originates from the top or the bottom of the extracted soil core. Using the metadata, we retrieved 575 KOs that show significant differential abundances across samples from desert vs. non-desert sampling locations (483 “up regulated,” 92 “down regulated”). We then utilized leapR’s functionality to retrieve enriched KEGG Pathways based on the KO abundance data. The pathway enrichment detected several enriched pathways, for example “sulfur cycle.” Utilizing map data from the US census bureau, the terra R package as well as the latitude and longitude annotations we are then able to visualize the pathway enrichment using the spatial_heatmap() function (Fig. 1C). A complete workflow can be found in the spatMicrobiome vignette that is part of spammR.

### 3.3 Integration of proteomic and lipidomic measurements in brain tissue

To show how spammR can be used to integrate multiple omics from the same sample, we leveraged data from Vandergrift *et al*. (2025) that measured lipidomics and proteomics from a tissue sample from rat brain. We used the retrieve_metaspace_data() to fetch the experimental data from the Metaspace portal (Palmer *et al*. 2017) and coordinates from the lipids, then downloaded the proteomics data from the original publication. The original lipid data, shown in Fig. 1D, spans the entire image, while the protein data (carved out as squares in the image) only represented a subset of the image. We therefore used the spat_reduce() function to align the two coordinate systems to map the average lipid expression to the regions that correspond to protein measurements, shown in Fig. 1E. Using this combined object, we could then build a correlation graph, shown in Fig. 1F, that identifies specific proteins correlated with a particular lipid of interest, in this case a phosphatidylserine identified as highly variable across the image (PS(28:6(10Z, 13Z, 16Z, 19Z, 22Z, 25Z)/12:0)). We then ran leapR analysis on the correlation results to identify a KEGG pathway significantly enriched (negatively correlated in this case) with the lipid, depicted in Fig. 1G.

## 4 Conclusion

Through spammR, we address multiple limitations in the current state-of-the-art tools for computational analysis of diverse spatial omics measurements with a suite of lightweight methods that can be broadly applied across mass spectrometry and sequence-derived measurements. spammR is built upon the existing Bioconductor framework for spatial omics measurements and can therefor interoperate with the ever-growing set of tool for spatial transcriptomics, but adds specific expansions to explicitly (a) account for issues that generally arise with mass spectrometry such as missingness and (b) align omics from diverse modalities in the same image.

We worked to include a diverse set of features that could be used in the spatial omics community, but made distinct choices about when to include functionality vs. provide an interface to others. For example, in the design of our impute_spe() we tried to address the importance of missing data (e.g. in data-dependent acquisition (DDA) (Wu and MacCoss 2002) proteomics data). In general, missing abundance for individual features does not necessarily equate to proteins not being expressed and can broadly be categorized into values missing (completely) at random (MCAR & MAR) and values missing not at random (MNAR). MCAR features can mostly be attributed to instrument errors and are truly random, whereas missingness of MAR features is dependent on the experimental setup and can often be traced back toknown instrument detection inaccuracies or differences between sample batches. However, both MAR and MCAR features tend to behave similarly, in that features are missing across the whole spectrum of intensities, making them more difficult to impute. On the other hand, MNAR values can often be traced back to technical limitations of the instrument, e.g. proteins of low expression not being detected due to intensities below the detection capabilities of the instrument resulting in left-censored missing data. Given the different nature of M(C)AR and MNAR values where missing values are likely to follow different intensity distributions, existing imputation strategies do not universally perform well for both types of missing values (Webb-Robertson *et al*. 2015, Lazar *et al*. 2016, Wei *et al*. 2018). No consensus for universal imputation strategies seems to be present in the scientific community. For example, assuming MNAR for observed missing values, Lui and Dongre propose that naïve single value imputation methods like SampMin (minimal value of the sample) can show good performance especially in downstream analyses like differential expression analysis whereas Harris et al. suggest the opposite(Harris *et al*. 2023, Kong *et al*. 2022, Liu and Dongre 2021). More recently, novel deep learning based approaches to imputation have demonstrated promising performance for both MAR and MNAR features in simulated mixed datasets (Webel *et al*. 2024, Segers *et al*. 2025), however with lower performance for datasets with increased MNAR fractions. Finally, more recent studies are suggesting to skip imputation all together (Harris *et al*. 2025). This highlights the fact there is no one universal solution to account for missing values and successful imputation strategies heavily depend on the dataset to be imputed. This is further complicated in spatial omics experiments, where cell-type specific proteins may only be expressed in one region of the tissue. To account for diverse use cases, impute_spe() currently includes eight imputation methods including spatially-aware imputation method that adapts k-nearest neighbor imputation to assign values to missing abundance data points based on molecular abundances only from samples that are spatially close to each other (rather than close to each other in expression value) with the hope that it can be used to benchmark and compare imputation algorithms in an experiment-specific context. Finally, considering that spammR is built on widely used data structures with a focus on interoperability with other R packages, users also have the ability to integrate other available imputation strategies from libraries like imputeLCMD (Lazar *et al*. 2022) or MsCoreUtils (Rainer *et al*. 2022) into their workflow.

We demonstrated the power of spammR for the analysis of a traditional spatial proteomics datasets using a spatial proteomics analysis of healthy human pancreatic tissue. Additionally, we showed that spammR is scale agnostic and can also be utilized on geospatial distance scale data by using a metagenome dataset highlighting the agnostic capabilities of spammR extending beyond the analysis of traditional multi-omic data. Lastly, we showed the power of integration using a small dataset that collects both lipidomics and proteomics from the same tissue.

In the future, we plan to continue expanding the functionality provided by spammR through the collection of larger, more complex datasets across tissues of interest. We aim to improve data processing through the incorporation of other spatial analysis such as the SpatialFeatureExperiment (Moses et al. 2023) and the direct incorporation of geoJSON format to link to tools such as quPath (Bankhead *et al*. 2017) or microscope vendor software. As multiomics datasets become more comprehensive, we also plan to improve our statistical methods for identification of functional pathways that account for measurements across omics and properly benchmark and assess imputation across omics data types.

## Data Availability

The data used by the examples presented here can be downloaded from either Figshare or Metaspace via the tutorials provided.
